# Supplementary material for: Temporal Changes in the Oxyhemoglobin Dissociation Curve of Critically Ill COVID-19 Patients
Source: J Clin Med. 2022 Jan 31;11(3):788. doi: 10.3390/jcm11030788 (PMC8836951; doi:10.3390/jcm11030788)
Supplement: Supplementary file 1 [file jcm-11-00788-s001.zip › jcm-1514666-supplementary.pdf]

Supplementary Material

Temporal changes in the oxyhemoglobin dissociation curve of critically ill COVID-19 patients are associated with clinical outcome.

AUTHORS: Samuele Ceruti, MD [1*], Bruno Minotti, MD [2*], Andrea Glotta, RN [1], Maira Biggiogero, PhD [3], Giovanni Bona, MD [3], Martino Marzano, MD [4], Pietro Greco, MD [5], Marco Spagnoletti, MD [5], Christian Garzoni, Ph.D [4+], Karim Bendjelid, MD, Ph.D [6+].

AFFILIATIONS:

1. Department of Critical Care, Clinica Luganese Moncucco, Lugano, Switzerland
2. Emergency Department, St. Gallen Cantonal Hospital, St. Gallen, Switzerland
3. Clinical Research Unit, Clinica Luganese Moncucco, Lugano, Switzerland
4. Department of Internal Medicine, Clinica Luganese Moncucco, Lugano, Switzerland
5. Emergency Department, Clinica Luganese Moncucco, Lugano, Switzerland
6. Intensive Care Division, Geneva University Hospitals, Geneva, Switzerland

[*] = these Authors contribute equally (co-first authorship)

[+] = these Authors contribute equally (co-last authorship)

CORRESPONDING AUTHOR:

Samuele Ceruti, MD, Department of Intensive Care Unit, Clinica Luganese Moncucco – Via Moncucco 10, 6900 Lugano, Switzerland, Phone: 0041 91 960 8108, E-mail: samuele.ceruti@moncucco.ch

Table S1. Patients' clinical, laboratory and arterial blood gas analysis characteristics.

	Unit	N	Mean	Median	SD	Min	Max	25th	75th
Clinical Characteristics									
Age	*Years*	32	62.06	64.50	11.73	29.00	81.00	54.50	71.75
BMI	*Kg/m²*	32	29.04	28.60	4.77	20.10	41.10	25.98	31.95
SAPS		32	43.28	42.00	16.31	13.00	75.00	29.75	53.00
SOFA		32	6.38	6.50	2.97	2.00	11.00	4.00	8.75
NEMS		32	31.31	36.00	9.26	18.00	42.00	19.25	39.00
ICU LOS	*days*	32	20.06	14.00	10.92	7.00	43.00	12.00	28.00
Laboratory									
White Cell	*mg/L*	32	9.28	7.75	5.95	2.80	35.00	5.65	11.13
Lymphocytes	*U/L*	32	1.27	0.80	2.27	0.20	13.20	0.53	1.00
ASAT	*µmol/L*	30	63.10	52.50	29.18	22.00	131.00	42.75	83.00
ALAT	*U/L*	31	47.65	42.00	26.69	18.00	123.00	28.00	56.00
Lactate	*U/L*	32	1.18	0.95	0.50	0.50	2.60	0.80	1.55
CRP	*U/L*	32	244.91	225.50	111.13	60.00	534.00	163.75	320.00
LDH	*G/L*	32	665.06	595.00	362.63	184.00	2291.00	481.75	770.50
Ferritin	*G/L*	31	2693.87	2101.00	2179.39	455.00	11000.00	1046.00	3857.00
Creatinine	*mmol/L*	32	126.47	88.50	106.73	53.00	521.00	72.75	122.50
Thrombocytes	*ng/mL*	32	241.72	223.50	89.13	110.00	458.00	190.50	299.75
Total Bilirubin	*µmol/L*	31	14.73	9.70	13.75	3.80	56.50	7.60	16.40
CK-MB	*G/L*	31	384.71	337.00	357.84	33.00	1680.00	82.00	562.00
Arterial blood gas analysis									
pH		3514	7.39	7.40	0.08	7.03	7.70	7.35	7.45
pO_2	*mmHg*	3514	82.60	77.40	25.26	40.10	367.00	67.30	91.50
pCO_2	*mmHg*	3508	48.08	46.50	12.13	20.20	109.00	39.70	53.90
HCO_3	*mmol/L*	3508	28.89	28.40	5.17	7.30	56.30	25.40	31.60
BE	*mmol/L*	3508	3.33	3.00	4.73	-18.00	27.90	0.30	5.90
SaO_2	*%*	3514	95.24	96.00	15.56	45.00	100.00	94.00	98.00
Hemoglobin	*g/dL*	3501	11.48	11.60	2.44	4.00	23.80	9.50	13.20
Hematocrit	*%*	3496	35.22	35.00	7.50	12.00	73.00	29.00	41.00
COHb	*%*	3514	1.37	1.40	0.46	0.00	3.50	1.10	1.60
MetHb	*%*	3513	0.92	1.00	0.36	0.00	3.80	0.60	1.20
Natrium	*mmol/L*	3503	146.85	147.00	6.13	105.00	165.00	143.00	151.00
Kalium	*mmol/L*	3501	4.03	4.00	0.79	1.50	9.00	3.70	4.30
Calcium	*mmol/L*	3504	1.14	1.14	0.08	0.17	1.55	1.10	1.19
Chlore	*mmol/L*	3503	109.86	110.00	6.64	100.00	140.00	106.00	114.00
Glucose	*mmol/L*	3514	8.95	8.70	3.15	1.60	112.00	7.10	10.30
Temperature	*°C*	2957	37.1	37.0	0.4	35.0	40.5	37.0	37.0
Lactate	*mmol/L*	3474	1.30	1.20	0.54	0.30	6.10	0.90	1.60

Table S2. All patients' p50s calculated according to Hill formula modified by Dash et al weighted by pH, pCO2, 2,3-BPG and Temperature, further stratified according to the early/late phase compared to ICU stay. According to Kolmogorov-Smirnov test, the analysis for each patients refused the hypothesis of normal data distribution.

	All p50s							Early p50s						Late p50s					
	N	Median	Min	Max	25th	75th	Var	N	Median	Min	Max	25th	75th	N	Median	Min	Max	25th	75th
**Patient 1**	135	20.70	14.50	27.74	19.21	23.75	10.18	18	20.16	15.92	22.44	19.26	21.27	11	18.78	15.60	20.42	16.93	20.22
**Patient 2**	17	17.33	15.72	20.02	16.63	18.61	1.42	12	17.29	15.72	19.15	16.38	18.19	10	17.91	16.22	20.02	16.86	18.90
**Patient 3**	46	19.25	12.69	25.10	17.87	21.66	7.69	9	18.39	16.88	25.09	17.12	23.01	11	19.21	15.19	23.48	17.97	20.91
**Patient 4**	200	19.52	13.28	40.72	17.85	21.20	8.61	14	19.30	17.01	24.25	18.05	20.88	9	18.53	16.77	23.35	17.82	19.53
**Patient 5**	47	17.87	10.83	25.31	16.55	19.65	7.29	11	19.68	15.58	23.02	18.62	22.23	16	17.65	14.38	18.88	16.57	18.38
**Patient 6**	50	19.61	15.29	22.88	18.71	20.47	2.50	12	19.50	15.29	22.06	18.79	20.38	16	19.36	16.72	21.93	18.56	20.23
**Patient 7**	44	19.86	13.81	27.90	18.24	22.75	8.40	11	22.00	13.81	27.90	20.32	22.74	17	18.26	14.76	20.43	17.21	19.21
**Patient 8**	24	19.03	15.57	23.56	17.44	20.12	4.06	12	20.06	16.53	23.56	18.87	21.75	11	17.85	15.57	19.97	16.85	19.57
**Patient 9**	99	17.51	11.14	28.58	14.97	19.04	14.58	12	21.47	15.80	27.39	18.03	22.93	12	17.11	15.16	18.81	16.73	17.59
**Patient 10**	54	20.12	13.65	25.70	18.81	21.92	4.84	11	19.55	17.13	25.70	18.82	23.18	15	19.76	16.87	23.66	18.36	21.74
**Patient 11**	151	21.55	12.46	36.23	19.35	25.02	21.15	18	19.18	12.46	23.52	17.22	21.75	28	28.88	21.99	36.23	25.03	31.08
**Patient 12**	90	19.42	14.34	37.84	17.10	23.41	31.87	13	20.27	17.12	24.47	18.19	21.68	12	16.17	14.34	18.43	15.23	17.24
**Patient 13**	58	20.82	15.56	30.15	18.92	22.19	8.52	16	23.46	19.52	30.15	21.77	26.29	10	18.02	15.56	21.39	16.38	19.26
**Patient 14**	47	17.86	11.42	24.22	16.61	19.29	7.12	16	18.47	14.14	24.22	17.08	21.19	14	17.31	11.42	20.00	16.36	18.06
**Patient 15**	114	19.48	14.84	28.32	18.51	20.74	5.46	18	23.71	16.92	28.32	21.00	25.70	13	19.70	17.44	21.87	18.92	20.53
**Patient 16**	196	20.84	12.42	29.70	19.33	22.69	8.77	16	19.74	16.26	29.68	18.18	21.29	11	16.15	12.42	18.59	15.20	17.69
**Patient 17**	223	19.92	12.62	36.90	18.51	22.10	10.73	18	22.22	15.98	26.63	19.04	23.82	13	18.59	15.40	24.22	16.90	19.76
**Patient 18**	37	26.38	18.53	33.14	23.56	28.58	11.53	18	27.45	18.53	33.14	24.60	29.26	18	25.47	22.51	31.89	22.77	26.61
**Patient 19**	232	22.03	14.91	33.68	20.63	23.89	9.07	17	23.68	17.38	30.41	20.27	26.86	16	22.14	14.91	30.28	20.45	25.05
**Patient 20**	23	19.40	17.01	22.65	18.74	20.32	1.89	10	19.38	17.01	21.18	18.06	21.11	13	19.40	17.68	22.65	18.87	20.18
**Patient 21**	140	18.59	13.26	28.44	17.40	20.95	7.08	17	21.09	15.59	28.44	19.02	24.30	13	18.52	13.26	19.43	17.46	18.78
**Patient 22**	57	18.16	14.88	53.32	16.87	19.69	6.89	18	19.64	16.13	28.03	17.75	22.68	16	17.95	15.73	20.20	16.72	18.26
**Patient 23**	86	21.08	15.42	30.95	19.32	22.93	8.20	18	18.54	15.42	30.07	16.71	20.70	17	22.27	18.50	26.84	20.38	24.63
**Patient 24**	199	20.80	14.53	33.84	19.14	22.76	9.53	14	23.17	16.25	33.84	19.50	29.20	16	19.71	16.70	25.18	18.91	21.17
**Patient 25**	23	18.47	15.80	28.56	16.77	20.13	8.77	12	19.43	16.97	28.56	17.87	22.25	8	16.64	15.80	19.20	16.00	19.01
**Patient 26**	113	20.59	15.74	39.50	18.87	22.87	14.11	13	18.38	17.54	19.47	17.79	19.06	15	18.50	16.56	20.99	17.49	19.67
**Patient 27**	14	19.52	17.91	24.65	18.54	22.04	4.83	11	19.58	17.91	24.65	18.59	23.42	10	19.37	18.18	24.65	18.54	23.46
**Patient 28**	199	21.24	15.40	29.81	19.89	22.64	5.87	11	17.84	15.52	20.15	16.96	18.86	14	20.75	18.01	23.77	19.65	21.38
**Patient 29**	48	20.88	16.36	23.56	19.78	21.95	2.82	16	20.44	16.36	23.01	18.79	21.59	16	20.36	17.49	22.68	18.79	21.46
**Patient 30**	34	18.43	14.12	24.46	16.97	19.81	3.97	18	19.10	14.12	24.46	17.92	20.33	15	18.11	15.88	20.06	16.48	19.17
**Patient 31**	36	18.75	15.41	22.09	18.17	19.84	1.86	13	18.16	15.41	19.86	17.16	18.63	11	19.30	18.10	22.09	18.45	19.98
**Patient 32**	121	24.03	15.80	38.71	21.26	27.67	24.84	18	23.88	17.75	28.55	19.13	26.19	17	31.20	25.49	37.97	29.04	32.65

Table S3. MANOVA analysis comparing pattern of p50 shift over time (right/left shift) and clinical, biological, laboratory variables. Table A: Levene's Test for clinical parameters and laboratory exams. Table B: "Between-Subjects effects" Test. (Type III Sum of Squares: a. R Squared = ,009 (Adjusted R Squared = -,028), b. R Squared = ,001 (Adjusted R Squared = -,036), c. R Squared = ,005 (Adjusted R Squared = -,032), d. R Squared = ,005 (Adjusted R Squared = -,032), e. R Squared = ,160 (Adjusted R Squared = ,129), f. R Squared = ,023 (Adjusted R Squared = -,013), g. R Squared = ,057 (Adjusted R Squared = ,022), h. R Squared = ,001 (Adjusted R Squared = -,035), i. R Squared = ,001 (Adjusted R Squared = -,036), j. R Squared = ,003 (Adjusted R Squared = -,034), k. R Squared = ,096 (Adjusted R Squared = ,063), l. R Squared = ,045 (Adjusted R Squared = ,009), m. R Squared = ,067 (Adjusted R Squared = ,032), n. R Squared = ,015 (Adjusted R Squared = -,022), o. R Squared = ,005 (Adjusted R Squared = -,032), p. R Squared = ,025 (Adjusted R Squared = -,011), q. R Squared = ,006 (Adjusted R Squared = -,030), r. Computed using alpha = ,05)

	Levene Statistic	df1	df2	P value
**Sex**	1.480	1	21.000	0.237
**Age**	0.240	1	26.361	0.628
**BMI**	0.017	1	23.576	0.899
**Hypertension**	0.321	1	21.000	0.577
**Diabetes**	0.036	1	29.941	0.850
**OSAS**	0.199	1	29.074	0.659
**COPD**	0.006	1	29.862	0.937
**PE**	0.841	1	29.177	0.367
**CVVHDF**	2.757	1	21.000	0.112
**ICU LOS**	0.049	1	26.167	0.827
**ICU outcome**	0.536	1	28.865	0.470
**SAPS**	4.549	1	26.996	0.042
**SOFA**	2.270	1	26.980	0.144
**NEMS**	0.760	1	25.374	0.392
**Total Bilirubin**	0.622	1	19.980	0.440
**ASAT**	0.006	1	26.734	0.939
**ALAT**	0.229	1	25.212	0.636
**Leucocytes**	2.714	1	10.566	0.129
**Lymphocytes**	1.283	1	10.401	0.283
**Thrombocytes**	0.012	1	26.794	0.913
**Creatinine**	0.639	1	21.421	0.433
**Lactates**	0.134	1	20.357	0.718
**CRP**	0.313	1	24.764	0.581
**LDH**	0.281	1	12.698	0.605
**Ferritin**	0.237	1	17.598	0.633
**CK**	0.424	1	19.995	0.522

Source		Type III Sum of Squares	df	Mean Square	F	P value	Partial Eta Squared	Noncent. Parameter
**Corrected Model**	Sex	.128[a]	1	0.128	1.480	0.233	0.047	1.480
	Age	32.781[a]	1	32.781	0.238	0.629	0.009	0.238
	BMI	.399[b]	1	0.399	0.016	0.901	0.001	0.016
	Hypertension	.057[c]	1	0.057	0.218	0.644	0.007	0.218
	Diabetes	.009[d]	1	0.009	0.036	0.850	0.001	0.036
	OSAS	.028[e]	1	0.028	0.199	0.658	0.007	0.199
	COPD	.001[f]	1	0.001	0.006	0.937	0.000	0.006
	PE	.327[h]	1	0.327	0.841	0.366	0.027	0.841
	CVVHDF	.355[i]	1	0.355	2.757	0.107	0.084	2.757
	ICU LOS	.384[k]	1	0.384	0.003	0.956	0.000	0.003

	ICU Outcome	.096[b]	1	0.096	0.536	0.470	0.018	0.536
	SAPS	40.948[c]	1	40.948	0.136	0.715	0.005	0.136
	SOFA	1.311[d]	1	1.311	0.144	0.707	0.005	0.144
	NEMS	383.160[e]	1	383.160	5.160	0.031	0.160	5.160
	Total Bilirubin	139.683[p]	1	139.683	0.689	0.414	0.025	0.689
	ASAT	36.425[h]	1	36.425	0.040	0.843	0.001	0.040
	ALAT	23.783[i]	1	23.783	0.031	0.862	0.001	0.031
	Leucocytes	23.338[f]	1	23.338	0.627	0.435	0.023	0.627
	Lymphocytes	9.033[g]	1	9.033	1.627	0.213	0.057	1.627
	Thrombocytes	1096.509[o]	1	1096.509	0.134	0.717	0.005	0.134
	Creatinine	5186.881[n]	1	5186.881	0.407	0.529	0.015	0.407
	Lactates	.024[j]	1	0.024	0.085	0.773	0.003	0.085
	CRP	36600.436[k]	1	36600.436	2.876	0.101	0.096	2.876
	LDH	176350.923[l]	1	176350.923	1.264	0.271	0.045	1.264
	Ferritin	8852517.738[m]	1	8852517.738	1.925	0.177	0.067	1.925
	CK	23532.444[q]	1	23532.444	0.174	0.680	0.006	0.174
**Intercept**	Sex	23.878	1	23.878	276.480	0.000	0.902	276.480
	Age	96299.678	1	96299.678	700.044	0.000	0.963	700.044
	BMI	22019.605	1	22019.605	862.395	0.000	0.970	862.395
	Hypertension	5.682	1	5.682	21.802	0.000	0.421	21.802
	Diabetes	4.009	1	4.009	16.056	0.000	0.349	16.056
	OSAS	0.778	1	0.778	5.568	0.025	0.157	5.568
	COPD	0.251	1	0.251	2.765	0.107	0.084	2.765
	PE	2.327	1	2.327	5.981	0.021	0.166	5.981
	CVVHDF	0.355	1	0.355	2.757	0.107	0.084	2.757
	ICU LOS	11020.009	1	11020.009	89.460	0.000	0.749	89.460
	ICU Outcome	15.846	1	15.846	88.480	0.000	0.747	88.480
	SAPS	47894.741	1	47894.741	158.816	0.000	0.855	158.816
	SOFA	1032.346	1	1032.346	113.561	0.000	0.808	113.561
	NEMS	23781.091	1	23781.091	320.237	0.000	0.922	320.237
	Total Bilirubin	5388.082	1	5388.082	26.559	0.000	0.496	26.559
	ASAT	104084.701	1	104084.701	114.789	0.000	0.810	114.789
	ALAT	62531.369	1	62531.369	80.939	0.000	0.750	80.939
	Leucocytes	2515.018	1	2515.018	67.586	0.000	0.715	67.586
	Lymphocytes	59.193	1	59.193	10.659	0.003	0.283	10.659
	Thrombocytes	1538187.268	1	1538187.268	187.530	0.000	0.874	187.530
	Creatinine	387946.054	1	387946.054	30.462	0.000	0.530	30.462

	Lactates	35.875	1	35.875	129.080	0.000	0.827	129.080
	CRP	1404135.332	1	1404135.332	110.344	0.000	0.803	110.344
	LDH	12980013.543	1	12980013.543	93.020	0.000	0.775	93.020
	Ferritin	213493372.634	1	213493372.634	46.421	0.000	0.632	46.421
	CK	4116600.444	1	4116600.444	30.408	0.000	0.530	30.408

$$\mathrm{P}_{50,\Delta pH} = P_{50,s} - 25.535(pH - pH_s) + 10.646(pH - pH_s)^2 - 1.764(pH - pH_s)^3$$

$$\mathrm{P}_{50,\Delta CO2} = P_{50,s} + 1.273 \times 10^{-1}\left(P_{CO2} - P_{CO2,S}\right) + 1.083 \times 10^{-4}\left(P_{CO2} - \; P_{CO2,S}\right)^2$$

$$\mathrm{P}_{50,\Delta DPG} = P_{50,s} + 795.63([DPG] - [DPG]_s) - 19660.89([DPG] - [DPG]_s)^2$$

$$\mathrm{P}_{50,\Delta T} = P_{50,s} + 1.435(T - T_s) + 4.163 \times 10^{-2}(T - T_s)^2 + 6.86 \times 10^{-4}(T - T_s)^3$$

$$\mathbf{P_{50}} = \boldsymbol{P_{50,s}}\left(\boldsymbol{P_{50,\Delta pH}} \;/\; \boldsymbol{P_{50,s}}\right)\left(\boldsymbol{P_{50,\Delta CO2}} \;/\; \boldsymbol{P_{50,s}}\right)\left(\boldsymbol{P_{50,\Delta DPG}} \;/\; \boldsymbol{P_{50,s}}\right)\left(\boldsymbol{P_{50,\Delta T}} \;/\; \boldsymbol{P_{50,s}}\right)$$

Figure S1. The polynomial expressions from Dash et al (12) refined the original Hill formula based on experimental data which provided appropriate shifts in S_{HbO2} with varying pH, pCO_2 and Temperature. In the first four expressions, standard p50 (p50s) was normalized according to different pCO_2, pH, 2,3-BPG and Temperature levels, keeping the other three variables fixed at their standard physiological values. The resulting polynomial expressions for $p50,_{\Delta pH}$, $p50,_{\Delta pCO2}$, $p50,_{\Delta BPG}$ and $p50,_{\Delta T}$ were fitted to the p50s, calculated according to Hill formula.

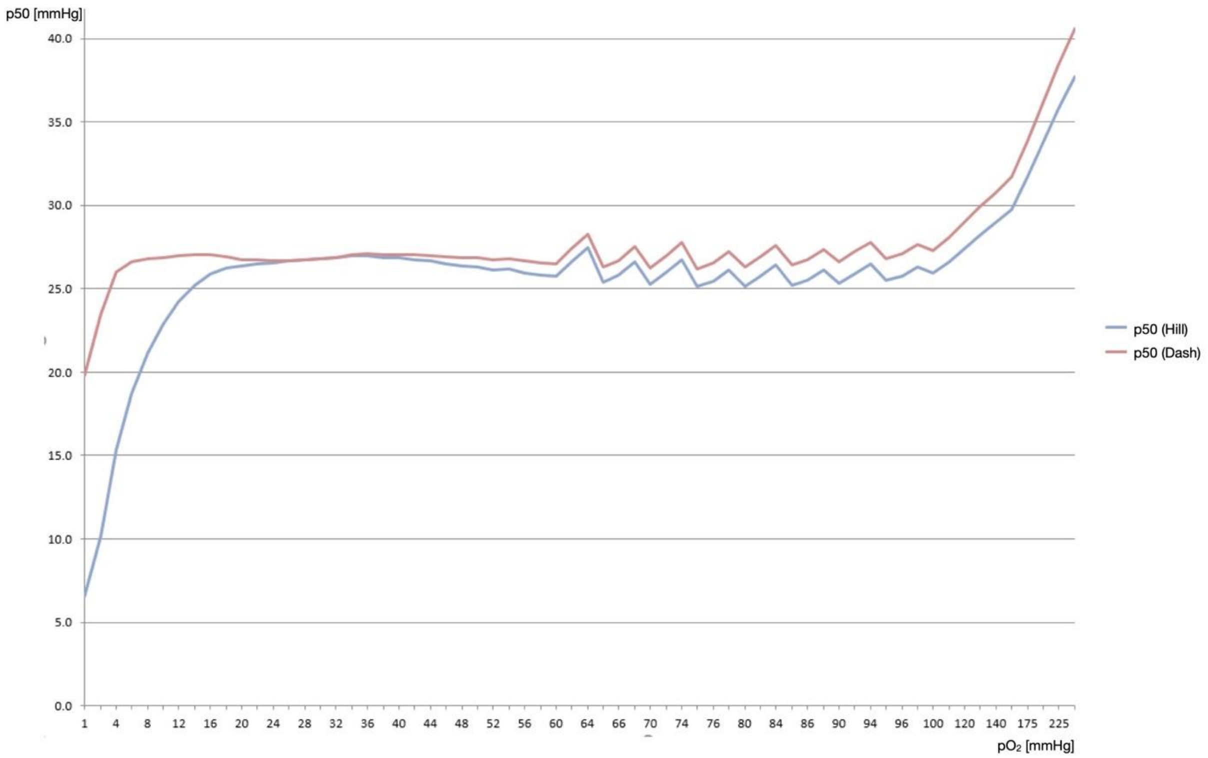

Figure S2. Graphic representation of p50 calculated according to standard Hill formula (orange) and with the coefficients weighted by pH, pCO_2, 2,3-BPG and Temperature, according to Dash et al (blue). Starting from a pO_2 of about 100 mmHg all both curve rises; this effect does not correspond to a real p50 shift, rather to a mathematical bias directly linked to the Hill formula itself.

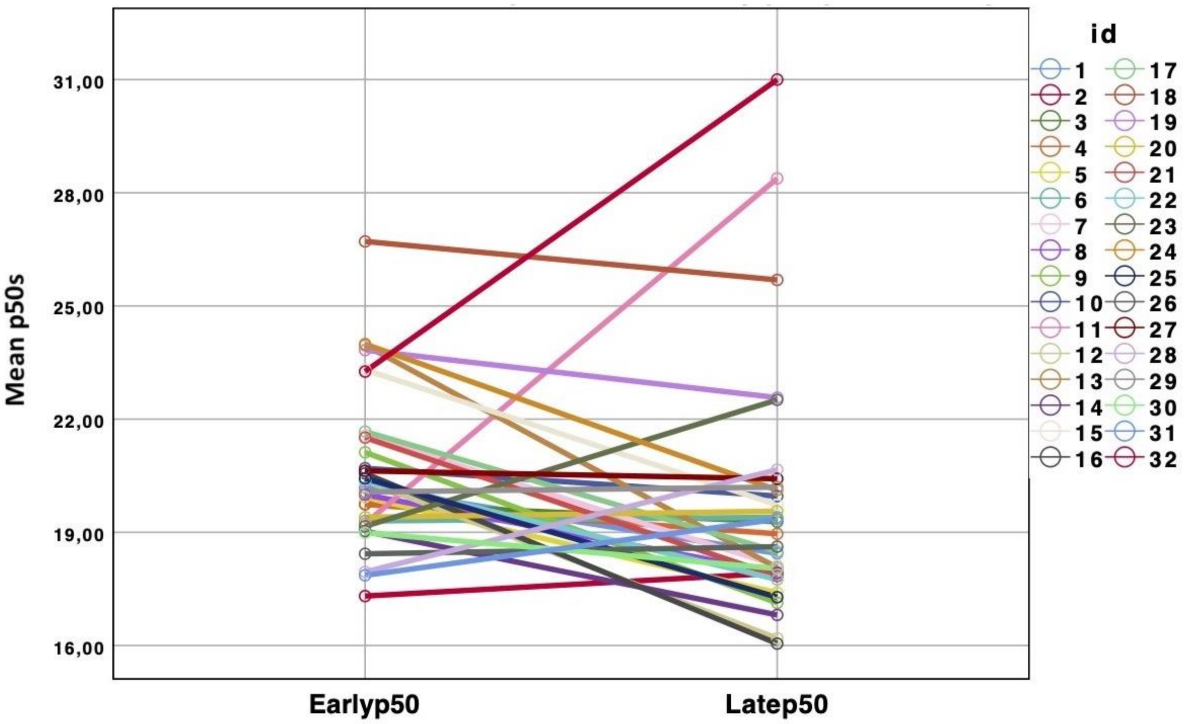

Figure S3. Means of early p50s and late p50s reported for each critically ill COVID-19 patient; the majority of patients show a reduction in p50 value over time (**left ODC shift**).

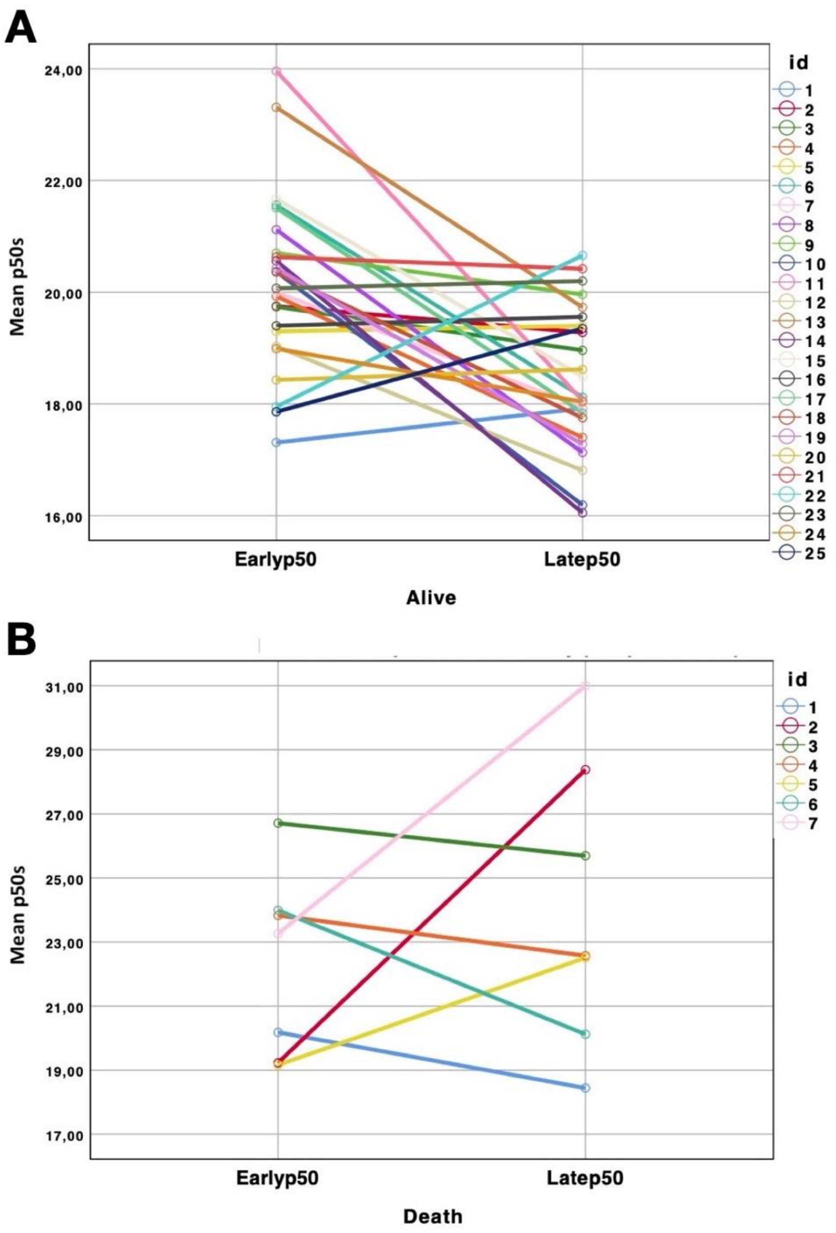

Figure S4. Means of early p50s and late p50s reported for each critically ill COVID-19 patient, stratified according to ICU discharge outcome as alive (**A**) or deceased (**B**). Most of the patients who survive show a reduction in p50 value over time (**left ODC shift**), while deceased patients show an increase in p50 value over time (**right ODC shift).** Neither Chi-square analysis nor MANOVA test revealed any correlation between p50 temporal pattern shift and ICU outcome.

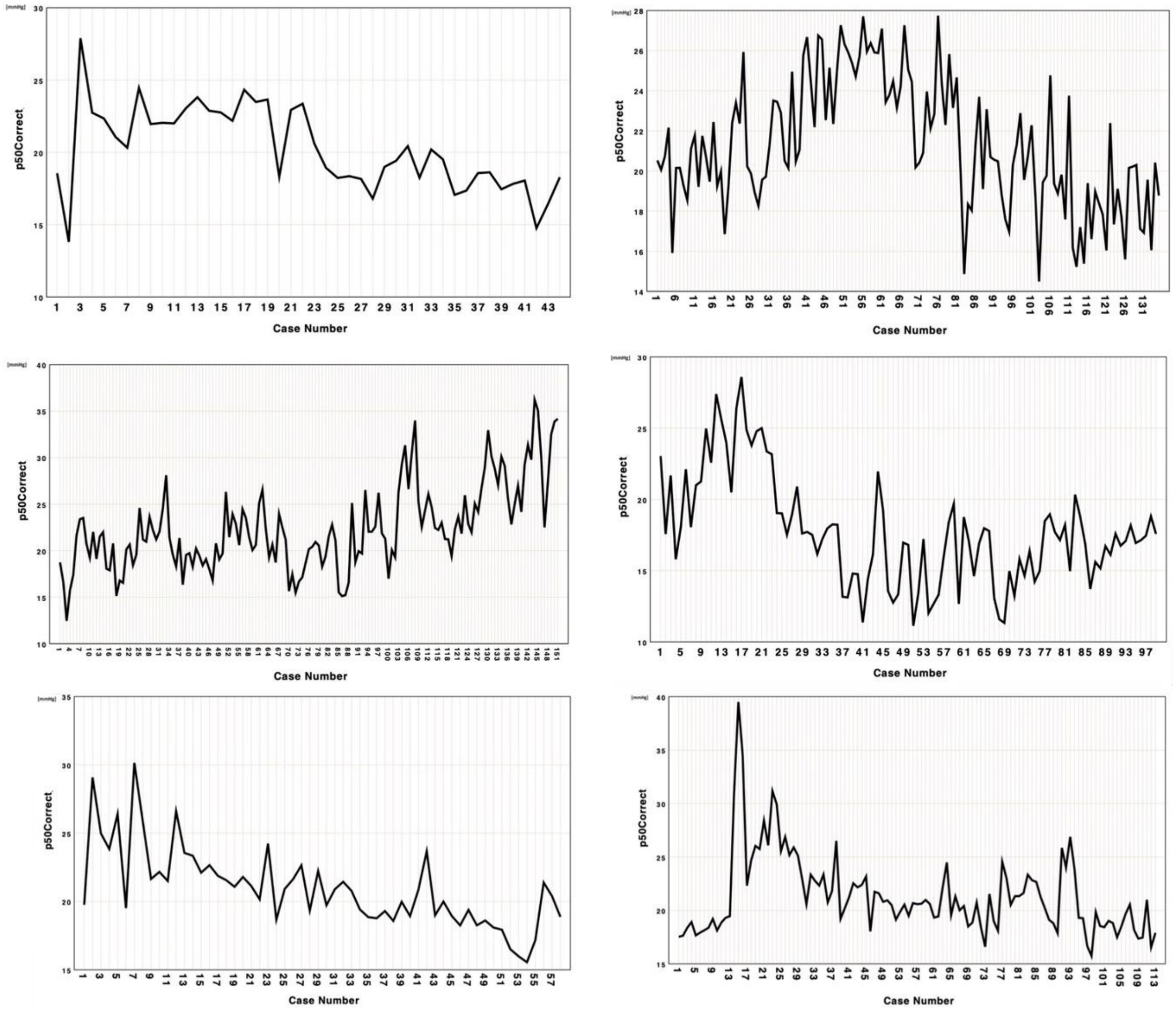

Figure S5. Examples of temporal evolution of p50 in 6 of 32 critically ill COVID-19 patients during their ICU stay; p50 constantly oscillated, with a consequent right/left ODC shift and Hb-O_2 affinity modification. "Case number" is referred as number of ABGAs for the same patient; "p50 correct" is intended as p50 calculated according to Hill formula modified by Dash et al.

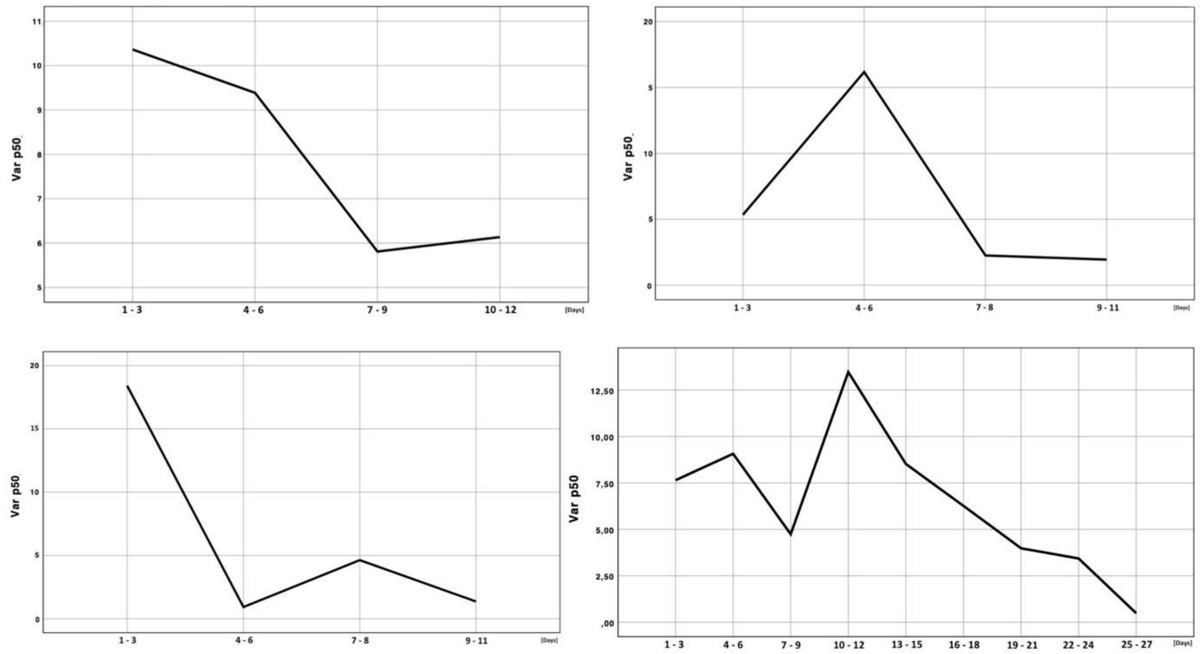

Figure S6. Temporal evolution of **p50 variance** over days in 4 of 32 critically ill COVID-19 patient during ICU stay; p50 Variance constantly modified itself, with a progressive reduction over time, going from high variance values at ICU admission to low variance at ICU discharge. “Days” is referred to days of ICU LOS; “Var p50”is referred to p50 Variance.

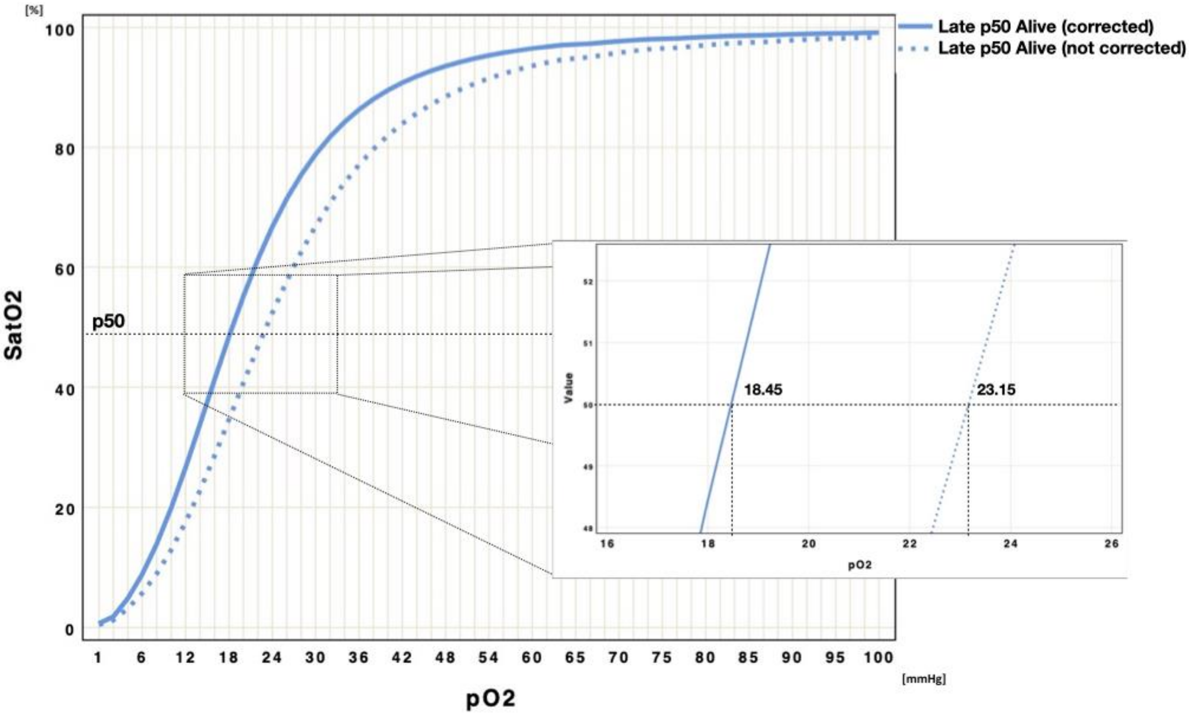

Figure S7. Graphic representation of Hb-O_2 affinity ODC, reporting the curve identified by the median of **late p50s** of patients discharged alive from the ICU, calculated according to the Hill formula modified by Dash (solid blue curve, 18.45 mmHg) and the median of early p50s calculated with the simple Hill formula (dashed blue curve, 23.15 mmHg).

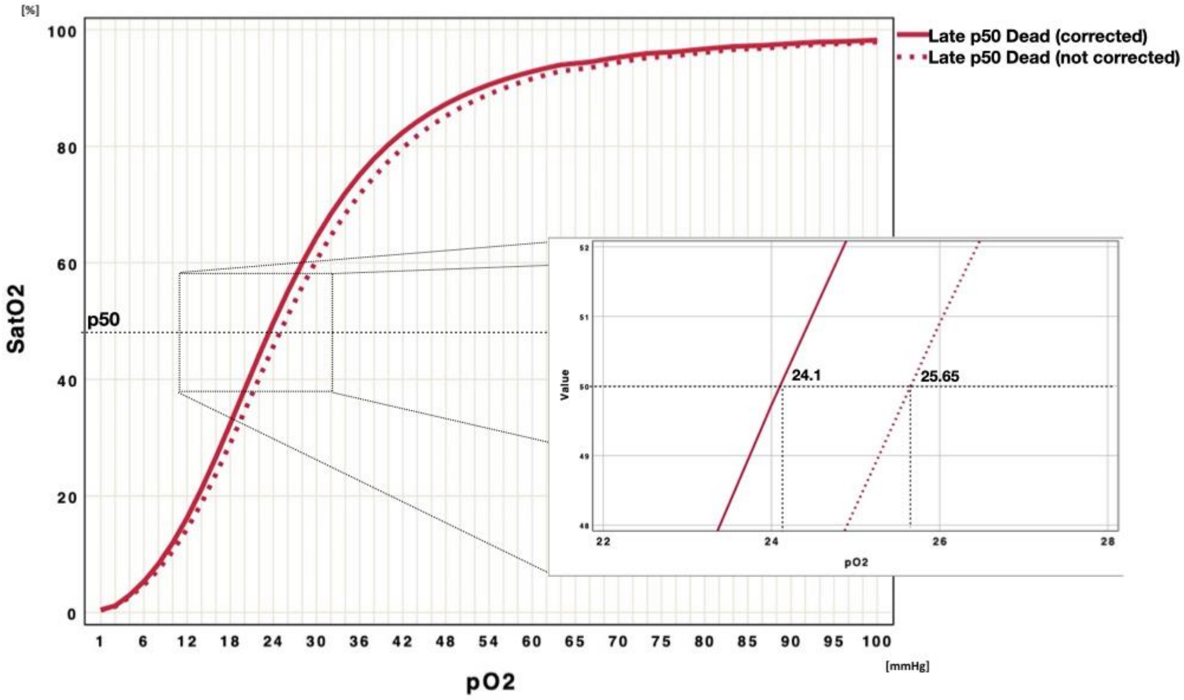

Figure S8. Graphic representation of Hb-O_2 affinity ODC, reporting the curve identified by the median of **late p50s** of patients discharged deceased from the ICU, calculated according to the Hill formula modified by Dash (solid red curve, 24.10 mmHg) and the median of late p50s calculated with the simple Hill formula (dashed red curve, 25.65 mmHg).
